# Comparative *in silico* characterization of virulence factors in major *Brucella* species reveals potential targets for vaccine and drug target discovery

**DOI:** 10.14202/vetworld.2026.1564-1580

**Published:** 2026-04-24

**Authors:** Arzu Özgen, Semiha Yalçın

**Affiliations:** 1 Department of Medical Laboratory Techniques, Vocational School of Health Service, Istanbul Gelişim University, Istanbul 34310, Türkiye; 2 Department of Microbiology, Milas Faculty of Veterinary Medicine, Muğla Sıtkı Koçman University, Muğla 48000, Türkiye

**Keywords:** bioinformatics, *Brucella*, lipopolysaccharide, lpxC, protein–protein interaction, type IV secretion system, vaccine targets, virulence factors

## Abstract

**Background and Aim:**

Brucellosis is a globally significant zoonotic disease that causes reproductive failure in animals and chronic infection in humans, resulting in substantial economic and public health burdens. The pathogenicity of *Brucella* species is largely mediated by virulence determinants such as the type IV secretion system and lipopolysaccharide biosynthetic machinery. Despite extensive studies on individual components, a comparative and integrative understanding of conserved virulence factors across major *Brucella* species remains limited. This study aimed to perform a comprehensive *in silico* characterization of key virulence proteins across representative *Brucella* species to identify potential targets for vaccine and antimicrobial development.

**Materials and Methods:**

Nine virulence-associated proteins (VirB3, VirB5, VirB7, WbkA, WbkB, WbkC, FabZ, gmd, and lpxC) from five major *Brucella* species were analyzed using bioinformatics approaches. Analyses included physicochemical characterization, subcellular localization prediction, conserved domain and motif identification, multiple sequence alignment, phylogenetic analysis, and protein–protein interaction network construction. Publicly available databases and tools such as NCBI, ProtParam, DeepLoc, MEME, MEGA11, and STRING were utilized.

**Results:**

Subcellular localization analysis revealed that VirB5 is extracellular and VirB7 is outer membrane-associated, whereas most other proteins were cytoplasmic or membrane-associated. Conserved motif analysis identified three shared motifs, particularly in VirB5 and WbkB, indicating functional conservation across species. Phylogenetic and sequence alignment analyses demonstrated high conservation of virulence proteins among the selected *Brucella* species. Protein-protein interaction networks highlighted VirB3, VirB5, VirB7, WbkC, FabZ, gmd, and lpxC as key interaction hubs. lpxC showed strong connectivity with lipid A biosynthesis proteins, suggesting its central functional role.

**Conclusion:**

This integrative *in silico* analysis identified conserved virulence proteins with potential translational relevance. VirB5 and VirB7 emerged as promising candidates for subunit vaccine development due to their extracellular or membrane localization and conserved motifs, while lpxC was identified as a potential antimicrobial target because of its central role in lipopolysaccharide biosynthesis. These findings provide a rational framework for future experimental validation and support the development of improved control strategies against brucellosis.

## INTRODUCTION

Brucellosis, also referred to as Mediterranean fever, Cyprus fever, Malta fever, and undulant fever [1], is one of the most economically important and widely distributed bacterial zoonoses worldwide [1–3]. The causative agents primarily colonize the reproductive system of domestic animals, particularly *Brucella abortus* and *Brucella melitensis*, and can lead to chronic, sometimes lifelong, debilitating infections in humans. These infections are characterized by severe clinical manifestations, including arthritis, fever, splenomegaly, and hepatomegaly [2].

The disease affects domestic animals such as cattle, goats, sheep, and pigs, causing abortion and metritis in females, and orchiepididymitis in males, ultimately leading to infertility, reduced fertility, and decreased milk production. Humans act as accidental hosts. Transmission occurs through direct contact with contaminated materials such as placenta, vaginal discharge, or aborted fetuses [1], inhalation of airborne bacteria, or ingestion of contaminated dairy products. Human-to-human transmission, although rare, may occur through organ transplantation, blood transfusion, or breastfeeding [4].

Bacteria of the genus *Brucella* belong to the phylum Proteobacteria, class Alphaproteobacteria, order Rhizobiales, and family Brucellaceae [1, 5]. The genus includes major pathogenic species such as *Brucella suis*, *B. melitensis*, and *B. abortus*, which are responsible for significant economic losses in livestock and are also associated with human infections [4]. Additional identified species include *Brucella canis*, *Brucella ovis*, *Brucella pinnipedialis*, *Brucella neotomae*, *Brucella microti*, *Brucella ceti*, *Brucella papionis*, *Brucella inopinata*, and *Brucella vulpis* [4, 5]. These organisms are non-spore-forming, non-encapsulated, non-motile, Gram-negative coccobacilli or short bacilli, and are facultative intracellular pathogens [6]. They possess the ability to adapt to the macrophage environment, survive, and replicate, as well as tolerate acidic pH and low oxygen conditions [1].

The genomes of all *Brucella* species exhibit similar size and structure, with an average genome size of approximately 3.29 Mb, consisting of two circular chromosomes. Chromosome I is approximately 2.11 Mb, while chromosome II is around 1.18 Mb, with G+C contents of 57.2% and 57.3%, respectively [6]. A major virulence factor of *Brucella* is lipopolysaccharide (LPS), which comprises lipid A, a core oligosaccharide, and an O-antigen chain [7]. Wild-type strains such as *B. melitensis*, *B. suis*, *B. abortus*, *B. neotomae*, *B. microti*, *B. ceti*, and *B. pinnipedialis* express smooth (S-type) LPS, whereas *B. ovis* and *B. canis* lack the O-polysaccharide and exhibit rough (R-type) LPS [8].

The lipid A component anchors LPS within the cell membrane, while the O-polysaccharide extends outward, determining the immunological properties of the bacterium. Diagnosis of brucellosis is based on detecting antibodies against the O-polysaccharide in human and animal sera [9]. LPS plays a crucial role in intracellular survival, exhibiting low endotoxicity, resistance to macrophage degradation, and evasion of host immune responses [10].

A total of nineteen genes essential for LPS synthesis and smooth phenotype expression have been identified in *B. melitensis* [11]. Most LPS biosynthesis genes are clustered in the wbo and wbk regions, with the wbk locus serving as the primary genetic region for O-polysaccharide synthesis. This region encodes enzymes involved in N-formylperosamine synthesis (*per*, *gmd*, *wbkC* genes), O-polysaccharide glycosyltransferases (*wbkA*, *wbkE* genes), ABC transporters (*wzt*, *wzm* genes), polyisoprenyl-phosphate N-acetylhexosamine-1-phosphate transferase (*wbkF* gene), and enzymes required for N-acetylamino sugar synthesis (*wbkD* gene) [12].

β-hydroxyacyl-acyl carrier protein dehydratase (FabZ) plays a key role in fatty acid biosynthesis during elongation cycles [13] and has also been associated with LPS formation [14]. The gene products of per, gmd, and wbkC are predicted to participate in 4-formamido-4,6-dideoxymannose synthesis [15]. Experimental studies have demonstrated that wbkC is essential for O-side-chain production, while wbkA is likely involved in O-side-chain polymerization due to its similarity to mannosyltransferases. The LPS structure of *Brucella* differs significantly from that of other Enterobacteriaceae such as *Escherichia coli* [15, 16], further contributing to its role as a virulence factor [17]. Genes involved in LPS synthesis, including *lpsA, lpsB/lpcC, lpxA, lpxB, lpxC, lpxD, lpxE, gmd, per, wbkA, wbkB, wbkC, wbpL, wbdA, wzm*, and *wzt*, are unique to *Brucella* isolates [18].

The type IV secretion system (T4SS), first identified in *B. suis* and encoded by the VirB operon, is highly conserved across all *Brucella* species. This system consists of twelve genes (*virB1*–*virB12*) responsible for secreting bacterial macromolecules [19, 20]. The expression of the VirB operon is regulated by the quorum-sensing regulator *vjbR* [21]. These proteins are essential for intracellular survival, enabling bacteria to establish a replication niche by interacting with the endoplasmic reticulum and acquiring its membrane [5]. The system is rapidly activated during intracellular infection, reaching peak activity within five hours and subsequently declining after the formation of replication vacuoles [19].

Despite extensive investigations into *Brucella* virulence mechanisms, most previous studies have focused on individual virulence factors or single species, resulting in a fragmented understanding of pathogenicity across the genus. In particular, key components such as the type IV secretion system and LPS biosynthesis pathways have been studied in isolation, with limited efforts to integrate their structural, functional, and interaction-based characteristics across multiple clinically relevant *Brucella* species. Furthermore, although several virulence-associated genes have been identified, their comparative conservation, subcellular localization, motif architecture, and interaction networks remain insufficiently explored in a unified analytical framework. This lack of integrative analysis restricts the ability to prioritize robust and broadly conserved targets for vaccine or antimicrobial development.

In addition, current vaccine strategies against brucellosis remain suboptimal, with limitations in cross-protection, safety, and differentiation of infected from vaccinated animals. Similarly, the identification of novel antimicrobial targets is hindered by an incomplete understanding of essential proteins within interconnected virulence pathways. Although bioinformatics tools are widely available, their combined application for comparative prioritization of conserved virulence determinants across multiple *Brucella* species has not been comprehensively addressed. Therefore, a systematic, multi-dimensional *in silico* evaluation of conserved virulence proteins is needed to bridge this gap and support rational target selection for translational research.

The present study was designed to address this gap by performing a comprehensive and integrative *in silico* characterization of selected virulence-associated proteins across major *Brucella* species. Specifically, the study aimed to comparatively analyze key proteins involved in the type IV secretion system and LPS biosynthesis, including VirB3, VirB5, VirB7, WbkA, WbkB, WbkC, FabZ, gmd, and lpxC, using multiple complementary bioinformatics approaches.

The objectives were to evaluate their physicochemical properties, predict subcellular localization, identify conserved domains and motifs, assess evolutionary relationships through phylogenetic analysis, and construct protein–protein interaction networks to determine functional connectivity. By integrating these analytical dimensions, the study sought to identify conserved and functionally significant proteins with potential translational relevance.

Ultimately, this work aims to provide a rational framework for prioritizing candidate proteins for subunit vaccine development and antimicrobial targeting, contributing to improved control strategies against brucellosis from a One Health perspective.

## MATERIALS AND METHODS

### Ethical approval

This study was conducted exclusively using publicly available genomic and proteomic datasets retrieved from the National Center for Biotechnology Information (NCBI) database and other open-access bioinformatics resources. No live animals, human participants, or biological samples were involved at any stage of the study. Therefore, ethical approval from an institutional animal care and use committee or human ethics committee was not required.

### Study period and location

The study was conducted using publicly available genomic and proteomic data retrieved from the NCBI database. All analyses were performed *in silico* between April 2024 and February 2026.

### Selection of *Brucella* species and virulence genes

**Figure 1:**
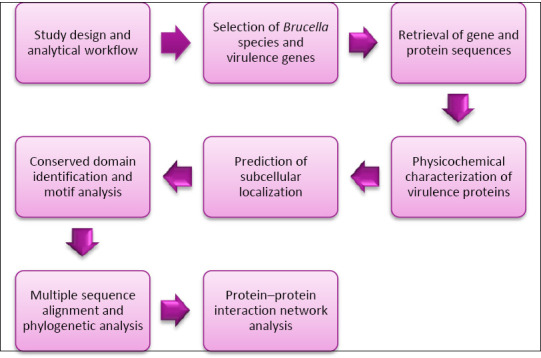
Schematic representation of the study design and analytical workflow.

The study design and analytical workflow are presented in Figure 1. The selection of *Brucella* species in this study was guided by biological, epidemiological, and phenotypic diversity considerations rather than random inclusion. The five selected species were chosen based on their established relevance to animal health and, in several cases, human infections, as well as their representation across multiple host species, including cattle, sheep, goats, pigs, and dogs.

Furthermore, both smooth and rough phenotypes were intentionally included to reflect structural variability in LPS composition and the associated immunological differences. This strategy ensured a broader representation of biological and epidemiological diversity across major *Brucella* lineages. The inclusion of both phenotypes also enhanced diversity in LPS structures, which are known to influence immune recognition, virulence, and serological behavior.

In this study, the objective was to explore conserved molecules with potential relevance as vaccine or therapeutic targets across *B. melitensis* bv. 1 str. 16M, *B. suis* 1330, *B. canis* ATCC 23365, *B. ovis* ATCC 25840, and *B. abortus* 2308.

The selection of nine virulence-associated genes (*virB3, virB7, virB5, wbkC, wbkB, wbkA, fabZ, gmd*, and *lpxC*) was based on their functional roles in key pathogenic mechanisms. In addition, the availability of sequence data for all selected species in open-access databases was considered as an important methodological criterion. These proteins were selected due to their established involvement in virulence, LPS biosynthesis, and host-pathogen interactions.

### Retrieval of gene and protein sequences

The genes (*virB3, virB7, virB5, wbkC, wbkB, wbkA, fabZ*, *gmd*, and *lpxC*) and their corresponding amino acid sequences from *B. melitensis* bv. 1 str. 16M, *B. suis* 1330, *B. canis* ATCC 23365, *B. ovis* ATCC 25840, and *B. abortus* 2308 were retrieved from the NCBI database (https://www.ncbi.nlm.nih.gov/gene/) (Accessed April 17, 2024).

### Physicochemical characterization of virulence proteins

The physicochemical properties of the nine selected virulence proteins were analyzed using the ProtParam tool available on the ExPASy server (http://web.expasy.org/protparam/) (Accessed April 17, 2024). The parameters evaluated included the number of amino acids, molecular weight, theoretical isoelectric point, instability index, and the grand average of hydropathicity (GRAVY).

### Prediction of subcellular localization

The predicted subcellular localization of the virulence proteins was determined using the DeepLoc-1.0 online server (https://services.healthtech.dtu.dk/services/DeepLoc-1.0/) (Accessed April 18, 2024). Protein sequences were submitted in FASTA format for analysis.

### Conserved domain identification and motif analysis

Information regarding conserved domains of the virulence proteins was obtained from the Conserved Domains Database (CDD) at NCBI (https://www.ncbi.nlm.nih.gov/guide/domains-structures/) (Accessed June 29, 2024). Protein sequences in FASTA format were submitted and analyzed at an E-value threshold of 0.01. The output displayed the best-scoring domain model along with its associated domain superfamily for each query region.

Conserved motif identification was performed using the Multiple Em for Motif Elicitation (MEME) server (Version 5.5.9) (https://meme-suite.org/meme/tools/meme) (Accessed Feb 19, 2026) for VirB3, VirB7, VirB5, WbkC, WbkB, WbkA, FabZ, gmd, and lpxC proteins.

### Multiple sequence alignment and phylogenetic analysis

Multiple sequence alignments were conducted using the amino acid sequences of the selected virulence proteins in Geneious Prime version 2024.0 (Biomatters; https://www.geneious.com). These analyses were used to evaluate the degree of similarity and divergence among virulence proteins.

Phylogenetic relationships were inferred using the Maximum Likelihood method with the Jones–Taylor–Thornton (JTT) matrix-based model [22]. The dataset consisted of 24 amino acid sequences, and evolutionary analyses were performed using Molecular Evolutionary Genetics Analysis (MEGA11).

### Protein–protein interaction network analysis

Protein–protein interaction networks were constructed using the STRING database (https://string-db.org/cgi/input?sessionId=bgzpUl8u2HmH) (Accessed May 03, 2024). The proteins VirB3, WbkB, WbkC, FabZ, gmd, and lpxC from *B. abortus* 2308 were selected to identify their interaction partners and functional connectivity.

### Software versions

The bioinformatics tools and software resources used in this study, along with their respective versions and access details, are summarized in Table 1.

**Table 1:** Bioinformatics tools and software resources used in the study.

Bioinformatic	Software name	Software version/ Accessed year	Software link
Physicochemical properties	Expasy Server	2024	http://web.expasy.org/protparam/
Prediction subcellular localization	DeepLoc-1.0	2024	https://services.healthtech.dtu.dk/services/DeepLoc-1.0/
Conserved domain	Conserved Domains NCBI	2024	https://www.ncbi.nlm.nih.gov/guide/domains-structures/
Motif analysis	MEME server	Version 5.5.9	https://meme-suite.org/meme/tools/meme
Multiple sequence alignments	Geneious Prime	Version 2024.0	https://www.geneious.com
Phylogenetic analysis	Molecular Evolutionary Genetics Analysis (MEGA)	MEGA11	https://www.megasoftware.net/
Protein–protein interaction network analysis	STRING	2024	https://string-db.org/cgi/input?sessionId=bgzpUl8u2HmH

## RESULTS

### Retrieval of gene and protein sequences

Gene and protein sequence information for *B. melitensis* bv. 1 str. 16M, *B. suis* 1330, *B. canis* ATCC 23365, *B. ovis* ATCC 25840, and *B. abortus* 2308 were obtained from the NCBI Gene database. The selected virulence-associated genes included *virB3*, *virB7*, *virB5*, *wbkC*, *wbkB*, *wbkA*, *fabZ*, *gmd*, and *lpxC*, together with the amino acid sequences of the proteins they encode. For each gene and its corresponding protein, key annotation details such as Gene ID, gene symbol, functional description, gene type, protein accession number (NCBI RefSeq), chromosomal location, and genomic reference sequence were compiled and organized. These features are summarized in Table 2 and served as the reference dataset for the subsequent motif, localization, phylogenetic, and protein–protein interaction analyses.

**Table 2:** The gene and protein features of virulence genes family members in Brucellaceae.

Species	Gene ID	Gene symbol	Gene description	Gene type	Protein accession (NCBI reference sequence)	Protein symbol	Chr. No.	Genomic sequence
*B. melitensis* bv. 1 str. 16M	29595902	BME_RS10325	type IV secretion system protein VirB3	protein coding	WP_002966512.1	VirB3	II	NC_003318.1
*B. suis* 1330	45053164	BR_RS10375						NC_004311.2
*B. canis* ATCC 23365	55591804	BCAN_RS10430						NC_010104.1
*B. ovis* ATCC 25840	45125486	BOV_RS10650						NC_009504.1
*B. abortus* 2308	3827980	BAB_RS26675						NC_007624.1
*B. melitensis* bv. 1 str. 16M	29595028	BME_RS10345	lipoprotein	protein coding	WP_002966516.1	VirB7	II	NC_003318.1
*B. suis* 1330	45053160	BR_RS10355						NC_004311.2
*B. canis* ATCC 23365	55591800	BCAN_RS10410						NC_010104.1
*B. ovis* ATCC 25840	45125482	BOV_RS10630						NC_009504.1
*B. abortus* 2308	NC_007624.1	BAB_RS26655						NC_007624.1
*B. melitensis* bv. 1 str. 16M	29595513	*virB5*	P-type DNA transfer protein VirB5	protein coding	WP_002966514.1	VirB5	II	NC_003318.1
*B. suis* 1330	45053162				WP_006191582.1			NC_004311.2
*B. canis* ATCC 23365	55591802				WP_004692616.1			NC_010104.1
*B. ovis* ATCC 25840	45125484				WP_006015000.1			NC_010104.1
*B. abortus* 2308	NC_007624.1				WP_006077544.1			NC_007624.1
*B. melitensis* bv. 1 str. 16M	29594278	*wbkC*	LPS biosynthesis N-formyltransferase WbkC	protein coding	WP_002963675.1	WbkC	I	NC_003317.1
*B. canis* ATCC 23365	55590265							NC_010103.1
*B. ovis* ATCC 25840	45123991							NC_009505.1
*B. abortus* 2308	3787272							NC_007618.1
*B. suis* 1330	45051620				WP_006189959.1			NC_004310.3
*B. melitensis* bv. 1 str. 16M	29594264	*wbkB*	protein WbkB	protein coding	WP_002963676.1	WbkB	I	NC_003317.1
*B. suis* 1330	45051621				WP_004691951.1			NC_004310.3
*B. canis* ATCC 23365	55590266				WP_004691951.1			NC_010103.1
*B. ovis* ATCC 25840	45123992				WP_006011447.1			NC_009505.1
*B. abortus* 2308	3787273				WP_002963676.1			NC_007618.1
*B. melitensis* bv. 1 str. 16M	29594259	*wbkA*	family 1 glycosyltransferase WbkA	protein coding	WP_006642693.1	WbkA	I	NC_003317.1
*B. suis* 1330	45051629							NC_004310.3
*B. canis* ATCC 23365	55590273							NC_010103.1
*B. ovis* ATCC 25840	45124000				WP_280176887.1			NC_009505.1
*B. abortus* 2308	3787283				WP_002969840.1			C_007618.1
*B. melitensis* bv. 1 str. 16M	29593646	*fabZ*	3-hydroxyacyl-ACP dehydratase FabZ	protein coding	WP_004683864.1	FabZ	I	NC_003317.1
*B. ovis* ATCC 25840	45124527				WP_004688426.1			NC_009505.1
*B. suis* 1330	45052194							NC_004310.3
*B. canis* ATCC 23365	55590834							NC_010103.1
*B. abortus* 2308	3788700				WP_002964280.1			NC_007618.1
*B. melitensis* bv. 1 str. 16M	29596094	*gmd*	GDP-mannose 4,6-dehydratase	protein coding	WP_004681750.1	gmd	II	NC_003318.1
*B. suis* 1330	45053472				WP_004687379.1			NC_004311.2
*B. canis* ATCC 23365	5592109				WP_004687379.1			NC_010104.1
*B. ovis* ATCC 25840	45125769				WP_006015606.1			NC_009504.1
*B. abortus* 2308	3788735				WP_002963680.1			NC_007618.1
*B. melitensis* bv. 1 str. 16M	29593377	*lpxC*	UDP-3-O-acyl-N-acetylglucosamine deacetylase	protein coding	WP_004684016.1	lpxC	I	NC_003317.1
*B. suis* 1330	45052435				WP_002964532.1			NC_004310.3
*B. canis* ATCC 23365	55591076							NC_010103.1
*B. ovis* ATCC 25840	45124767							NC_009505.1
*B. abortus* 2308	3788783							NC_007618.1

### Physicochemical properties of proteins

The physicochemical properties of nine virulence proteins were predicted and analyzed via using PortParam to obtain their number of amino acids, molecular weight, theoretical isoelectric point, instability coefficient, and the grand average of hydropathicity (GRAVY) (Table 3).

**Table 3:** Predicting results of the physicochemical properties of virulence proteins.

Protein accession (NCBI Reference sequence)	Number of aa	Mw (Da)	pI	Instability index	GRAVY
WP_002966512.1 (VirB3)	116	13081.61	11.41	36.22 (stable)	0.309 (hydrophobic)
WP_002966516.1(VirB7)	57	5931.01	8.98	44.71 (unstable)	0.182 (hydrophobic)
WP_002966514.1 (VirB5)	238	26810.35	5.61	48.87 (unstable)	–0.599 (hydrophilic)
WP_006191582.1 (VirB5)	238	26860.41	5.47	48.72 (unstable)	–0.589 (hydrophilic)
WP_004692616.1 (VirB5)	238	26811.33	5.47	49.43 (unstable)	–0.599 (hydrophilic)
WP_006015000.1 (VirB5)	238	26800.31	5.61	49.68 (unstable)	–0.595 (hydrophilic)
WP_006077544.1 (VirB5)	238	26829.40	5.67	49.68 (unstable)	–0.604 (hydrophilic)
WP_002963675.1 (WbkC)	259	29128.48	5.89	38.31 (stable)	–0.033 (hydrophilic)
WP_006189959.1 (WbkC)	259	29110.45	5.89	38.60 (stable)	–0.023 (hydrophilic)
WP_002963676.1 (WbkB)	284	32322.82	5.52	49.07 (unstable)	–0.119 (hydrophilic)
WP_004691951.1 (WbkB)	284	32381.93	5.72	48.98 (unstable)	–0.107 (hydrophilic)
WP_006011447.1(WbkB)	284	32425.00	5.52	49.28 (unstable)	–0.090 (hydrophilic)
WP_006642693.1(WbkA)	376	42431.96	9.36	35.57 (stable)	–0.089 (hydrophilic)
WP_280176887.1 (WbkA)	376	42419.91	9.36	33.66 (stable)	–0.103 (hydrophilic)
WP_002969840.1 (WbkA)	376	42446.03	9.42	35.45 (stable)	–0.090 (hydrophilic)
WP_004683864.1 (FabZ)	157	17186.95	5.60	26.64 (stable)	0.088 (hydrophobic)
WP_004688426.1 (FabZ)	157	17187.94	5.78	25.41 (stable)	0.059 (hydrophobic)
WP_002964280.1 (FabZ)	157	17213.97	5.60	25.41 (stable)	0.071 (hydrophobic)
WP_004681750.1 (gmd)	356	40681.44	5.71	33.05 (stable)	–0.545 (hydrophilic)
WP_004687379.1 (gmd)	356	40667.42	5.71	32.82 (stable)	–0.544 (hydrophilic)
WP_006015606.1 (gmd)	356	40731.52	5.71	33.64 (stable)	–0.541 (hydrophilic)
WP_002963680.1 (gmd)	362	41062.55	5.98	32.55 (stable)	–0.395 (hydrophilic)
WP_004684016.1 (lpxC)	286	30866.93	4.94	33.21 (stable)	0.013 (hydrophilic)
WP_002964532.1 (lpxC)	286	30852.91	4.94	33.08 (stable)	0.012 (hydrophilic)

Mw = Molecular weight, aa = Amino acid, pI = Isoelectric point, GRAVY = Grand average of hydropathicity

### Prediction of subcellular localization

Using the DeepLocPro 1.0 server, prokaryotic proteins can be assigned to six localities: cytoplasm, cytoplasmic membrane, periplasm, outer membrane, cell wall and surface, and extracellular space. The potential subcellular location predictions of virulence proteins were obtained for lpxC, gmd, FabZ, WbkA, and WbkC as cytoplasmic, WbkB, and VirB3 as cytoplasmic membrane, VirB7 as outer membrane, and VirB5 as extracellular Table 4.

**Table 4:** Prediction of the probabilities of subcellular locations for virulence proteins in *Brucella* sp.

Protein	Localization	Cell wall and surface	Extracelular	Cytoplasmic	Cytoplasmic membrane	Outer membrane	Periplasmic
VirB3	Cytoplasmic Membrane	0.0003	0.0023	0.0172	0.8849*	0.0935	0.0018
VirB7	Outer Membrane	0.0001	0.0019	0.0005	0.0652	0.9315*	0.0008
VirB5	Extracellular	0.0008	0.8865*	0.0007	0.0019	0.0879	0.0222
WbkC	Cytoplasmic	0.0000	0.0014	0.9810*	0.0077	0.0098	0.0001
WbkB	Cytoplasmic Membrane	0.0009	0.0288	0.2660	0.6487*	0.0517	0.0039
WbkA	Cytoplasmic	0.0005	0.0036	0.7018*	0.2400	0.0535	0.0006
FabZ	Cytoplasmic	0.0000	0.0001	0.9864*	0.0090	0.0042	0.0003
gmd	Cytoplasmic	0.0004	0.0062	0.9327*	0.0186	0.0313	0.0107
lpxC	Cytoplasmic	0.0000	0.0006	0.9749*	0.0003	0.0239	0.0002

Higher subcellular location scores are indicated by asterisks.

### Conserved domain identification and motif analysis

**Figure 2:**
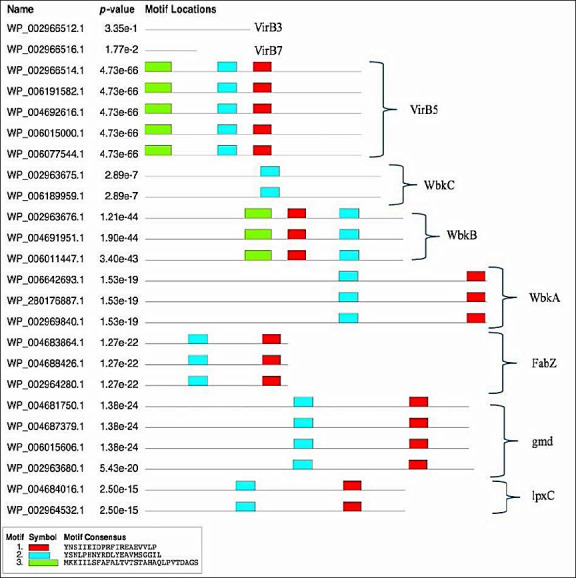
Schematic representation of the conserved motif of virulence proteins of *Brucella melitensis* bv. 1 str. 16M, *Brucella suis* 1330, *Brucella canis* ATCC 23365 and *Brucella abortus* 2308.

Information on conserved domains of virulence proteins was obtained from the CDD at NCBI and is presented in Table 5. The online MEME server was used to identify conserved motifs for the VirB3, VirB7, VirB5, WbkC, WbkB, WbkA, FabZ, gmd, and lpxC proteins and showed Table 6 and Figure 2.

**Table 5:** Conserved domain of virulence proteins in *Brucella* spp.

Protein	Domain name	Accession	Description	Interval	E-value
VirB3	VirB3	COG3702	Type IV secretory pathway, VirB3 component	18-113	1.65 × 10-31
VirB7	YcfL	COG5633	Uncharacterized conserved protein YcfL	1-43	3.59 × 10-5
VirB5	VirB5	TIGR02791	P-type DNA transfer protein VirB5	1-217	4.42 × 10-98
WbkC	FMT_core	cd08369	Formyltransferase, catalytic core domain	9-182	4.90 × 10-62
WbkB			No domains identified		
WbkA	GT4_MtfB like	cd03809	glycosyltransferases MtfB, WbpX, and similar proteins	6-362	2.65 × 10-100
FabZ	fabZ	PRK00006	3-hydroxyacyl-ACP dehydratase FabZ	6-150	7.22 × 10-86
gmd	Gmd	COG1089	GDP-D-mannose dehydratase [Cell wall/membrane/envelope biogenesis]	4-343	0
lpxC	LpxC	COG0774	UDP-3-O-acyl-N-acetylglucosamine deacetylase [Cell wall/membrane/envelope biogenesis]	4-279	2.97 × 10-157

**Table 6:** The identified motif of virulence proteins in *Brucella* spp.

Motif	Symbol	E-value	Sites	Width	Motif Consensus
1	red	2.2e-098	20	20	YNSIIEIDPRFIREAEVVLP
2	blue	2.4e-100	22	21	YSNLPHNYRDLYEAVMSGGIL
3	green	4.0e-094	8	29	MKKIILSFAFALTVTSTAHAQLPVTDAGS

### Multiple sequence alignment and phylogenetic analysis

**Figure 3:**
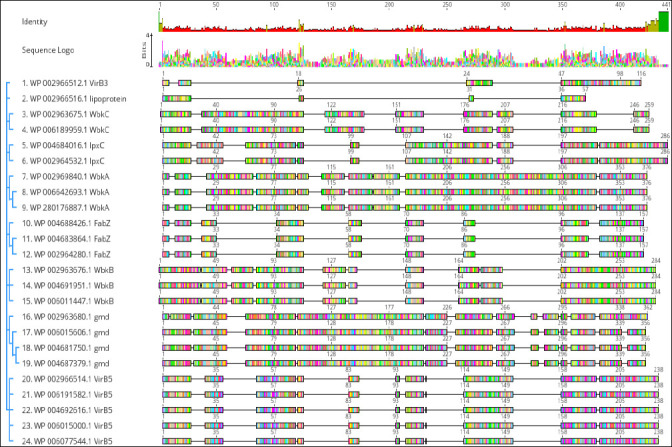
Multiple sequence alignment of VirB3, VirB7, VirB5, WbkC, WbkB, WbkA, FabZ, gmd, and lpxC proteins of *Brucella melitensis* bv. 1 str. 16M, *Brucella suis* 1330, *Brucella canis* ATCC 23365, *Brucella ovis* ATCC 25840 and *Brucella abortus* 2308. Geneious version 2024.0 created by Biomatters. Available from https://www.geneious.com.

**Figure 4:**
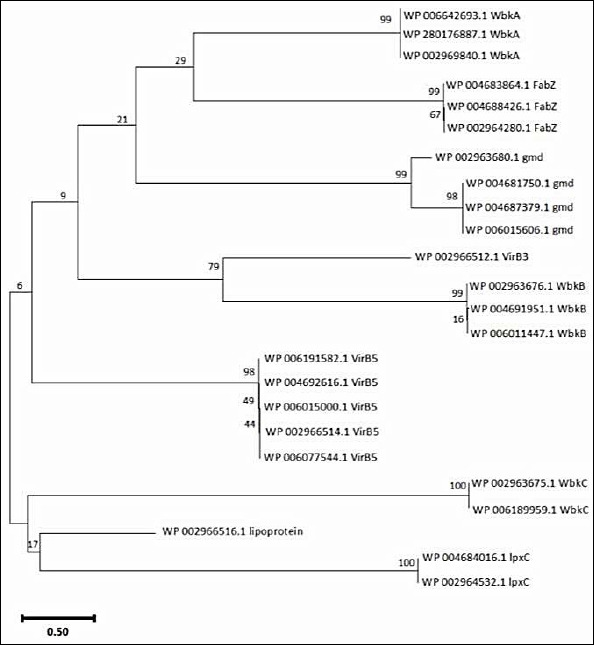
Phylogenetic analysis of virulence proteins of *Brucella* sp. The phylogenetic tree was generated using the amino acid sequences of selected virulence proteins by applying the Maximum Likelihood method and the JTT matrix-based model.

When the resulting VirB3, VirB7, VirB5, WbkC, WbkB, WbkA, FabZ, gmd, and lpxC proteins multiple sequences alignment was visualized in Geneious Prime version 2024.0 (Figure 3) conserved and distinct amino acids were identified in the *B. melitensis* bv. 1 str. 16M, *B. suis* 1330, *B. canis* ATCC 23365, *B. ovis* ATCC 25840 and *B. abortus* 2308 virulence proteins sequences. The evolutionary history was inferred by using the Maximum Likelihood method and Jones–Taylor–Thornton (JTT) matrix-based model [22]. The tree with the highest log likelihood (-6454.69) is shown. The percentage of trees in which the associated taxa clustered together is shown above the branches. Initial tree(s) for the heuristic search were obtained automatically by applying Neighbor-Join and BioNJ algorithms to a matrix of pairwise distances estimated using the JTT model, and then selecting the topology with the superior log likelihood value. The tree is drawn to scale, with branch lengths measured in the number of substitutions per site. This analysis involved 24 amino acid sequences. There were 424 positions in the final dataset. Evolutionary analyses were conducted in MEGA11 [23] (Figure 4).

### Interactive analysis of virulence proteins of *B. abortus* 2308

**Figure 5:**
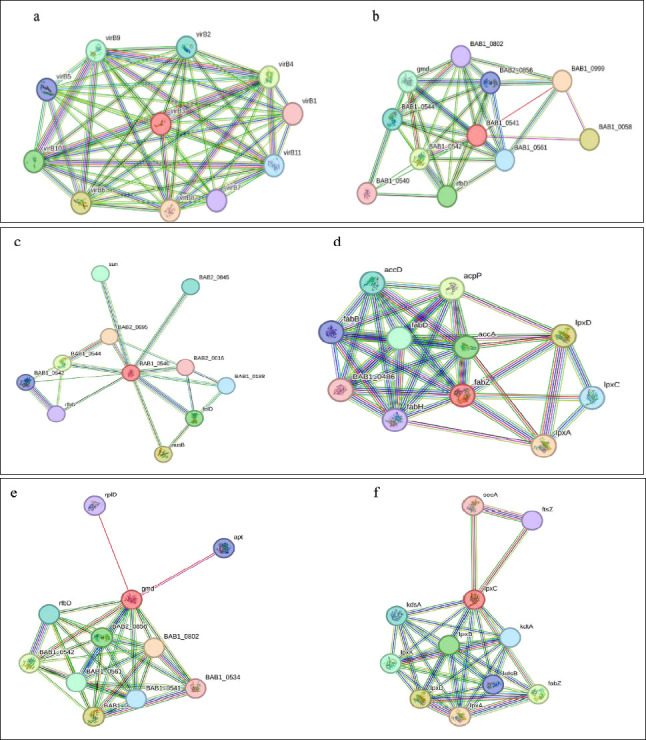
Interactive network of virulence proteins of *Brucella abortus* 2308. a) Interactive network of virulence protein VirB3, b) Interactive network of virulence protein WbkB, c) Interactive network of virulence protein WbkC, d) Interactive network of virulence protein FabZ, e) Interactive network of virulence protein gmd, and f) Interactive network of virulence protein lpxC.

The interactive protein study unfolds the numerous interacting partners of VirB3, WbkB, WbkC, FabZ, gmd, and lpxC (Figure 5) and their interactive protein partners (Table 7). The VirB2-4-5-6-8-9-10-11 had very high interactions with reference protein VirB3 (Figure 5a). The reference VirB3 protein was also highly associated with the VirB1 and VirB7. The BAB1_0999, BAB1_0058, BAB1_0542, RfbD, gmd, BAB1_0544, BAB1_0561, and BAB2_0856 had high interactions with reference protein WbkB (BAB1_0541) (Figure 5b). BAB1_0999 is a conserved hypothetical protein that shows glycosyltransferase activity and functions in the LPS biosynthetic process. The activities of BAB1_0058, BAB1_0542, RfbD, gmd, BAB1_0544, BAB1_0561, and BAB2_0856 proteins are summarized in Table 7. In addition, BAB1_0802 ve BAB1_0540 had medium interactions with reference protein WbkB [24, 25, 26]. The BAB2_0695 had very high interactions with reference protein WbkC (BAB1_0540) (Figure 5c). BAB2_0695 has NAD-dependent epimerase/dehydratase activity [24]. NusB, BAB1_0544, FolD, Sun, BAB2_0845, BAB1_0188, BAB1_0542, RfbD, whose activities are summarized in Table 7, had high interactions with reference protein WbkC. The lpxA, lpxD, AcpP, AccA, FabD, AccD, lpxC, FabB, FabH, and BAB1_0486 had very high interactions with reference protein FabZ (Figure 5d). The BAB1_0802 had a very high interaction with the reference protein gmd (Figure 5e). Also, BAB1_0544, BAB1_0542, BAB2_0856, BAB1_0561, RfbD, BAB1_0541, Apt, RplD, and BAB1_0534 had high interactions with the reference protein gmd. The lpxA, lpxD, FabZ, lpxB, lpxK, and KdsA had very high interactions with the reference protein lpxC (Figure 5f). Also, the KdtA, KdsB, FtsZ, and SecA had high interactions with the reference protein lpxC.

**Table 7:** Interactive network of virulence proteins of *Brucella abortus* 2308.

Virulence protein	Predicted functional partners name	Predicted functional partners
VirB3	VirB8	Type IV secretion system protein
	VirB6	Type IV secretion system protein
	VirB4	Type IV secretion system protein
	VirB10	Glutelin: Proline-rich region:Bacterial conjugation TrbI-like protein;
	VirB9	Type IV secretion system CagX conjugation protein
	VirB2	K03197 type IV secretion system protein
	VirB11	Type IV secretion system protein
	VirB5	Attachment mediating protein
	VirB7	Type IV secretion system protein
	VirB1	SLT domain
WbkB	BAB1_0999	Conserved hypothetical protein
	BAB1_0058	Esterase/lipase/thioesterase, active site
	BAB1_0542	ATP/GTP-binding site motif A (P-loop):ABC transporter:AAA ATPase.
	RfbD	ABC transporter, family 2: Acriflavin resistance protein.
	*gmd*	Short-chain dehydrogenase/reductase SDR
	BAB1_0544	DegT/DnrJ/EryC1/StrS aminotransferase
	BAB1_0561	Mannose-6-phosphate isomerase, type II
	BAB2_0856	Mannose-6-phosphate isomerase, type II
	BAB1_0802	Bacterial sugar transferase.
	BAB1_0540	Formyl transferase, N-terminal.
WbkC	BAB2_0695	NAD-dependent epimerase/dehydratase.
	NusB	Antitermination protein
	BAB1_0544	DegT/DnrJ/EryC1/StrS aminotransferase.
	FolD	Tetrahydrofolate dehydrogenase/cyclohydrolase
	Sun	SAM (and some other nucleotide) binding motif
	BAB2_0845	SAM (and some other nucleotide) binding motif
	BAB1_0188	Methionine synthase
	BAB1_0542	ATP/GTP-binding site motif A (P-loop)
	RrfbD	ABC transporter, family 2
	BAB2_0016	Condensation domain.
FabZ	*lpxA*	Bacterial transferase hexapeptide repeat.
	*lpxD*	UDP-3-O-acylglucosamine N-acyltransferase.
	AcpP	Acyl carrier protein (ACP).
	AccA	Acetyl-CoA carboxylase, alpha subunit.
	FabD	Acyl transferase domain.
	AccD	Acetyl-CoA carboxylase carboxyl transferase, beta subunit.
	*lpxC*	UDP-3-0-acyl N-acetylglucosamine deacetylase.
	FabB	Beta-ketoacyl synthase.
	FabH	β-ketoacyl-ACP synthase III
	BAB1_0486	Protein kinase: Beta-ketoacyl synthase.
*gmd*	BAB1_0802	Bacterial sugar transferase.
	BAB1_0544	DegT/DnrJ/EryC1/StrS aminotransferase.
	BAB1_0542	ATP/GTP-binding site motif A (P-loop).
	BAB2_0856	Mannose-6-phosphate isomerase, type II.
	BAB1_0561	Mannose-6-phosphate isomerase, type II.
	RfbD	ABC transporter, family 2.
	BAB1_0541	Perosamine synthetase WbkB.
	Apt	Adenine phosphoribosyltransferase.
	RplD	Ribosomal protein L4/L1e.
	BAB1_0534	Polysaccharide biosynthesis protein CapD.
*lpxC*	*lpxA*	Bacterial transferase hexapeptide repeat.
	*lpxD*	UDP-3-O-acylglucosamine N-acyltransferase.
	FabZ	Thioesterase superfamily.
	*lpxB*	Glycosyl transferase, family 19.
	*lpxK*	Tetraacyldisaccharide-1-P 4’-kinase.
	KdsA	DAHP synthetase I/KdsA superfamily.
	KdtA	Three-deoxy-D-manno-octulosonic-acid transferase, N-terminal.
	KdsB	Acylneuraminate cytidylyltransferase.
	FtsZ	Cell division protein FtsZ.
	SecA	SecA protein.

ABC = ATP-binding cassette, ATP/GTP = Adenosine Triphosphate/Guanosine Triphosphate, DAHP = 3-Deoxy-D-arabinoheptulosonate 7-phosphate, NAD = Nicotinamide Adenine Dinucleotide, SAM = S-Adenosyl methionine, SDR = The short-chain dehydrogenases/reductase, SLT = Soluble Lytic Transglycosylases, UDP-3-O-acylglucosamine = Uridine diphosphate-3-O-acylglucosamine

## DISCUSSION

### Physicochemical characteristics of selected virulence proteins

*Brucella* species are facultative intracellular pathogens known to cause serious zoonotic diseases around the world [27]. The virulence of these pathogens depends on their ability to evade or modulate the host immune response, as well as their capacity to survive and replicate within a vacuole derived from the endoplasmic reticulum [28].

Proteins with an instability index above 40 indicate that the protein is unstable and has a short half-life [29]. The GRAVY value is commonly used to evaluate the hydrophilicity and hydrophobicity of proteins. Moreover, the GRAVY value ranges between −2 and 2, where a negative or positive value indicates that the protein possesses hydrophilic or hydrophobic properties, respectively [30]. VirB3, VirB7, and FabZ proteins are predicted to be hydrophobic, whereas VirB5, WbkC, WbkB, WbkA, gmd, and lpxC proteins are predicted to be hydrophilic.

### Conserved domains and motif architecture of virulence-associated proteins

Considering the properties of the conserved domains of VirB3, VirB7, VirB5, WbkC, WbkA, FabZ, gmd, and lpxC proteins, respectively, the VirB3 protein contains the VirB3 superfamily covering amino acids 18-113, the VirB7 protein contains the putative periplasmic lipoprotein domain between amino acids 1-43, and the VirB5 protein contains the VirB5_like domain covering amino acids 1-217 [31]. There are main genes in the *wbk* locus, and one of the proteins encoded by these genes, the WbkC protein, contains the formyltransferase catalytic core domain encompassing amino acids 9-182 [17]. When the conserved domain for the WbkA protein was examined, it was observed that it belongs to glycosyltransferase family 4 (GT4) between amino acids 6-362 [32]. The gmd protein contains the GDP-D-mannose dehydratase domain between amino acids 4-343 [33]. The FabZ protein contains the 3-hydroxyacyl-ACP dehydratase FabZ domain between amino acids 6-150 [24]. The lpxC protein contains the UDP-3-O-acyl-N-acetylglucosamine deacetylase domain between amino acids 4-279.

Protein sequence motifs are patterns of residues that exhibit distinct structural and functional characteristics. They have been identified using multiple sequence and structural alignment methods as conserved signatures typical of protein families [34]. An important aspect of biological sequence characterization is the identification of motifs and domains [35]. Three conserved motifs were obtained:”YNSIIEIDPRFIREAEVVLPV” (Motif 1, red), “YSNLPHNYRDLYEAVMSGGIL” (Motif 2, blue), and “MKKIILSFAFALTVTSTAHAQLPVTDAGS” (Motif 3, green). *B. melitensis* bv. 1 str. 16M, *B. suis* 1330, *B. canis* ATCC 23365, *B. ovis* ATCC 25840, and *B. abortus* 2308 had all three motifs in *VirB5* and WbkB virulence proteins. Motif 1 (red) and Motif 2 (blue) were detected in WbkA, FabZ, gmd, and lpxC virulence proteins. No motif was detected in VirB3 and VirB7 virulence proteins. All virulence proteins except VirB3, VirB7, and WbkC have the “YNSIIEIDPRFIREAEVVLPV” motif represented in Motif 1 (red). The conserved motif analysis and the phylogenetic tree are consistent with each other.

### Functional significance of protein–protein interaction networks

Understanding biological processes requires a well-organized understanding of the physical interactions between proteins [36]. Proteins are essential molecules in living organisms, performing a variety of critical functions within cells, such as signaling, regulating metabolism, and maintaining cell structure [37]. Proteins interact with one another, regulating each other’s functions and binding together to fulfill their roles in biological processes [36]. Investigating protein–protein interactions not only enhances our understanding of vital cellular functions but also plays a significant role in the development of disease treatments and the design of drugs [37].

Virulence factors are essential for pathogen infection, making the interaction of virulence factors particularly important. LPS is an essential virulence factor of *Brucella* [5]. The virulence factors lpxA, lpxC, KdsB, and KdsA are involved in different stages of lipid A biosynthesis, an essential component of the LPS structure [38].

### lpxC as a potential antimicrobial target in *Brucella*

Although lpxC has been extensively validated as a drug target in other Gram-negative pathogens such as *E. coli*, *Pseudomonas aeruginosa*, and *Acinetobacter baumannii* [39, 40], its role as a therapeutic target in *Brucella* has not been widely addressed. Protein-protein interaction analyses have revealed that lpxC interacts significantly with a variety of proteins. Inhibition of lpxC may influence multiple interconnected components of the LPS biosynthetic pathway rather than definitively destabilizing the entire machinery. To the best of our knowledge, lpxC has not been widely emphasized as a rational drug target in *Brucella*, and our *in silico* network, motif, and localization data provide a complementary perspective for anti-*Brucella* drug development.

### Vaccine relevance of type IV secretion system-associated proteins

The interactive network analysis in this study highlights that inhibiting one of the proteins such as VirB3, VirB5, VirB7, WbkC, WbkA, FabZ, gmd, or lpxC may affect several functionally associated proteins and biological processes rather than guaranteeing a direct cascade failure. This synergistic vulnerability suggests that these proteins are not only potential drug targets but may also represent promising vaccine candidates when surface-exposed or secreted (e.g., VirB5, VirB7). Moreover, the conserved motifs identified, particularly those shared across *Brucella* species, may provide a basis for designing multi-epitope subunit vaccines aimed at inducing broad protective immunity [19, 41–44].

*Brucella* T4SS proteins play critical roles in the intracellular life cycle of the bacterium and may serve as targets for the immune system. In the literature, studies on VirB proteins have mostly focused on single antigens. For example, Tan *et al*. [45] reported that recombinant VirB5 protein could serve as a strong antigen for the serological diagnosis of bovine brucellosis, demonstrating high specificity (97.8%). Similarly, Pollak et al. [46] showed that immunization with VirB7 in combination with VirB9 in mice and dogs elicited a Th1-type immune response and reduced splenic bacterial load by approximately 1 log.

### Comparative significance of VirB3, VirB5, and VirB7 in target prioritization

VirB3 is located in the cytoplasmic membrane, which limits its immune accessibility. The importance of VirB3 does not stem from sequence conservation or surface exposure, but rather from its central functional role within the T4SS and its strong connections with multiple VirB components in the protein–protein interaction network. This network centrality supports prioritizing VirB3 as a functional drug target rather than as a primary vaccine candidate.

By comparison, although VirB5 and VirB7 are not absolute central hubs in the interaction network, they are functionally associated with the T4SS and show significant interactions with VirB3, indicating biological relevance within the system. When their immune accessibility advantage is considered, these two proteins emerge as complementary and potentially synergistic candidates for subunit vaccine design.

### Broad-spectrum vaccine implications of the VirB5-VirB7 combination

Our findings indicate that the combined evaluation of VirB5 and VirB7 may provide a rational basis for a potential broad-spectrum subunit vaccine perspective. VirB5 was shown to harbor conserved motifs across all *Brucella* species in this study and to be extracellularly localized, while VirB7 was predicted to be an outer membrane-associated lipoprotein with strong interactions within the T4SS network. These features suggest that both proteins could represent promising core immunogens for a multi-epitope vaccine strategy rather than isolated antigen selection.

Nevertheless, considering the motif distribution observed in our study, the likelihood of stronger cross-protective potential appears particularly high for five *Brucella* spp., where VirB5 motifs were consistently conserved. These conclusions are consistent with previous reports highlighting conserved outer membrane and T4SS proteins as cross-protective antigens and align with recent reviews emphasizing multi-antigen subunit or motif-based vaccines as promising next-generation strategies [19, 20, 41–44, 46, 47].

To the best of our knowledge, the VirB5-VirB7 combination has received limited attention in previous *Brucella* vaccine studies, and our findings support its consideration as a potential vaccine target. It should be noted that the distinct contribution of the present study does not arise from the individual bioinformatic tools employed, which are widely used in molecular analyses, but from their integrative interpretation across multiple complementary analytical dimensions. This combined evaluation enabled a comparative prioritization of conserved virulence factors rather than a purely descriptive characterization, thereby providing a more rational and evidence-guided framework for translational vaccine and drug target exploration.

### One Health relevance of conserved virulence factors across *Brucella* species

Since the *Brucella* species included in this study are known to infect different hosts, the conserved proteins identified here may have relevance across multiple species rather than being limited to a single host. From a One Health perspective, recognizing shared virulence mechanisms between animal and human infections can contribute to more integrated prevention and control strategies. Although these findings are based on computational analyses, the cross-species conservation observed suggests a possible value for broader zoonotic surveillance, vaccine research, and antimicrobial target exploration in both veterinary and human health contexts.

## CONCLUSION

This study provides a comprehensive *in silico* evaluation of key virulence-associated proteins (VirB3, VirB5, VirB7, WbkC, WbkB, WbkA, FabZ, gmd, and lpxC) across multiple *Brucella* species, highlighting their conserved structural, physicochemical, and functional characteristics. The results demonstrated that several proteins, particularly VirB5, VirB7, WbkA, FabZ, gmd, and lpxC, exhibit favorable stability profiles and conserved domains associated with essential biological functions such as type IV secretion system activity, LPS biosynthesis, and intracellular survival. Motif analysis revealed shared conserved sequences across species, supporting their evolutionary conservation and potential functional importance. Additionally, protein–protein interaction network analysis identified strong connectivity among virulence factors, indicating their coordinated role in pathogenicity.

From a practical perspective, these findings suggest that selected virulence proteins may serve as promising candidates for the development of novel diagnostic, therapeutic, and preventive strategies. In particular, *lpxC* emerges as a potential antimicrobial target due to its central role in lipid A biosynthesis and its extensive interaction network. Similarly, extracellular and outer membrane-associated proteins such as VirB5 and VirB7 demonstrate strong potential as vaccine candidates due to their accessibility to the host immune system with VirB5 further distinguished by its conserved motif architecture, which supports its potential as a promising vaccine target.. The identification of shared motifs further supports the feasibility of designing multi-epitope or subunit vaccines with broader cross-protective potential across *Brucella* species.

A major strength of this study lies in the integrative analytical approach, combining physicochemical characterization, conserved domain identification, motif analysis, subcellular localization prediction, phylogenetic relationships, and protein–protein interaction networks. This multi-dimensional framework enables a more rational prioritization of virulence factors compared to single-method analyses. Furthermore, the inclusion of multiple *Brucella* species representing diverse hosts enhances the translational relevance of the findings within a One Health context.

However, this study has several limitations. The conclusions are based solely on computational predictions and database-derived sequences, which may not fully reflect biological complexity under in vivo conditions. Functional validation of the identified targets, including gene knockout studies, protein expression analyses, and immunogenicity assessments, was not performed. In addition, host-specific immune responses and environmental factors influencing protein expression were not considered.

Future research should focus on experimental validation of the prioritized targets, particularly lpxC, VirB5, and VirB7, using in vitro and in vivo models. Structural biology approaches, such as protein crystallography and molecular docking studies, may further clarify their suitability as drug targets. Moreover, vaccine development studies incorporating conserved motifs into multi-epitope constructs should be explored to assess cross-protective immunity across different *Brucella* species. Integrating genomic, proteomic, and immunological data will further strengthen the translational applicability of these findings.

In conclusion, this study provides a systematic and integrative framework for identifying conserved virulence-associated proteins in *Brucella* spp. and highlights their potential relevance in antimicrobial and vaccine development. The findings contribute to a deeper understanding of *Brucella* pathogenesis and support the advancement of targeted control strategies within a One Health perspective.

## DATA AVAILABILITY

The supplementary data can be available from the corresponding author upon a reasonable request. Figure S1-S9: Prediction of subcellular localization of virulence protein of *Brucella* spp.; Table S1: Motif analysis parameters of virulence protein of *Brucella* spp.; Figure S10: Multiple sequence alignment of virulence protein of *Brucella* spp.; Protein sequences of virulence proteins of *Brucella* spp.; Table S2-7: Node degree of virulence proteins of VirB3, WbkB, WbkC, FabZ, gmd, and lpxC; Figure S11-16: Predicted functional partners of VirB3, WbkB, WbkC, FabZ, gmd, and lpxC; Figure S17: STRING Settings parameters.

## AUTHORS’ CONTRIBUTIONS

AO and SY: Conceptualization, investigation, writing – original draft, validation, writing – review and editing. Both authors have read and approved the final manuscript.
